# Study of the potential adverse effects caused by the dermal application of *Dillenia indica* L. fruit extract standardized to betulinic acid in rodents

**DOI:** 10.1371/journal.pone.0217718

**Published:** 2019-05-31

**Authors:** Flávia S. Fernandes, Gustavo S. da Silva, Alexandre S. Hilel, Ana C. Carvalho, Karina V. T. Remor, Aline D. Schlindwein, Luiz A. Kanis, Daniel F. Martins, Maicon R. Kviecinski

**Affiliations:** Universidade do Sul de Santa Catarina, Postgraduate Program in Health Sciences, Palhoça, Santa Catarina, Brazil; University of Iowa, UNITED STATES

## Abstract

This study aimed to evaluate the potential adverse effects of the dermal administration of *Dillenia indica* Linnaeus (*D*. *indica*) fruit extract in healthy rodents; the extract was standardized to betulinic acid. In the initial phase, the acute effects were evaluated on the skin application site of a single extract dose. A skin irritation test was performed in male *Wistar* rats (n = 8/group) receiving the extract (50–150 mg/mL) with betulinic acid (0.5–1.5%, respectively). A photosensitivity test was performed in male BALB/c mice (n = 6/group) receiving the extract (150 mg/mL). Afterwards, other BALB/c mice (n = 20, male:female, 1:1) were used to assess the systemic alterations caused by 14 daily repeated doses (150 mg/mL) by monitoring the effects on mortality, body morphology, behavior, nutrition status, neuromotor reactions, organ morphology and weight, and blood tests. At this time, 0.5 mg/mL clobetasol was used as the positive control. The skin irritation index suggested that negligible skin irritation had occurred, even when the extract was applied to the rat skin at 150 mg/mL. However, the extract acted as a photosensitizer on mouse skin, showing a photosensitizing activity close to that of 10 mg/mL 5-methoxypsoralen. Repeated doses caused no mouse mortality, aggressiveness, piloerection, diarrhea, convulsions, neuromotor alterations or nutrition status changes. The mouse organ weights did not change, and the mice did not have alterations in their blood compositions. Clobetasol caused a reduction in the mononuclear leukocyte numbers. In general, the data suggest that the extract was safe in healthy rodents but indicate that caution should be taken with the photosensitizing activity; in addition, this activity should be further explored as it may be useful for phototherapeutic drug development.

## Introduction

This work was originated from an interest in natural products that could be sources of innovative drugs for the treatment of immune-mediated dermatological disorders, such as psoriasis. Psoriasis is currently the most prevalent autoimmune disease worldwide [1,2]. The available treatment has a low efficacy and many side effects [3,4]. Therefore, a survey was initially carried out with people who worked with folk medicine and integrative therapies. This survey included more than one hundred participants that included elderly and pastoral health groups in the southern region of Brazil. Based on this survey, the species *Dillenia indica* Linnaeus (*D*. *indica*) was chosen to be investigated more thoroughly [5].

*D*. *indica* is a tree species that belongs to the Dilleniaceae family. This plant is native to Asian tropical forests and grows in central and southern India, reaching some regions of China and Oceania [6–9]. This plant was introduced in Brazil in the nineteenth century and is well adapted, mainly in the coastal region [10]. Its fruit are remarkable, hard and large, with a diameter of approximately four centimeters [7,11]. One of the most famous popular names used for *D*. *indica* is elephant-apple, a name given because the fruit are relished by elephants, which are important seed dispersers for this tree. These fruit are used for medicinal purposes and in many cuisines [6,7]. Most of the traditional uses are associated with anti-inflammatory purposes [12].

A large number of scientific studies on *D*. *indica* have shown that extracts derived from this species have various properties, such as anti-inflammatory, antimicrobial, antidiabetic, hypolipidemic, antidiarrheal, antioxidant and antileukemic effects [7,10,12–18]. Additionally, previous studies have shown that *D*. *indica* fruit have an interesting anti-inflammatory potential. Some phytochemical studies have shown that *D*. *indica* fruit contain primary and secondary metabolites and are a rich source of glucosides of triterpenoids (betulinic acid mostly), flavonoids, tannins and other less abundant constituents [6,18]. Despite the large number of previous studies, most of them have not evaluated standardized extracts or isolated substances, which made it difficult to infer the role of any particular constituent.

Taking these facts into account, our research group had previously performed a study evaluating the healing effect of *D*. *indica* fruit extracts that were standardized to betulinic acid on ultraviolet (UV) radiation-induced psoriasis-like wounds in rats. The most promising results were obtained for the ethyl acetate extract, which contained the highest content of betulinic acid [10]. Because of these promising results, clinical studies to further examine the utility of this extract would seem to be needed. However, good preclinical justifications are necessary, in terms of not only efficacy but also safety, prior to proceeding with clinical studies. The clinical studies evaluate extracts and substances as drugs, which are administered by specific routes. This context differs from the dietary intake of *D*. *indica* fruit.

To make progress in the development of a new drug to dermally treat psoriasis, we were inspired by the fact that both *D*. *indica* and betulinic acid have already been extensively explored in the literature, with many reports of beneficial effects, but that safety studies are still very scarce. The safety evaluations of a new drug include risk evaluations and the possibility to minimize these risks. This consists of the data acquisition of toxicology evaluations, side effect monitoring and prevention [19,20]. The purpose of the current study was to evaluate the potential adverse effects of the dermal administration of *D*. *indica* fruit extract standardized to betulinic acid in healthy rodents.

## Materials and methods

### Extraction and standardization to betulinic acid

The fruit of *D*. *indica* were harvested in the gardens of Universidade do Sul de Santa Catarina (Unisul) in Tubarão, Brazil. Professor Jasper José Zanco (from the same university) evaluated the plant’s authenticity. A voucher specimen was deposited at Herbarium *Laelia purpurata* in Unisul (voucher number SRS5103). This study followed all the biological biodiversity rules. Fresh ripe fruit were milled and maintained in shake-assisted macerators in ethyl acetate (Vetec, USA, ≥99%) at 1:2 (w/v) for 2 days, and this extraction was repeated three times. The solvent was eliminated under reduced pressure after the extractions were complete. The extraction performance was calculated in terms of dried extract yield (%), based on the starting material mass. It was approximately 0.70 ± 0.03%. Betulinic acid (107.60 ± 5.10 mg/g) was quantified using high-performance liquid chromatography (HPLC) performed in a Shimadzu workstation (Shimadzu, Japan) [18,21]. This technique was validated, and the calibration curve was constructed using betulinic acid standard solutions (Sigma-Aldrich Cat. 91466, ≥ 97.0%). Precisely weighed samples of extract were dissolved in HPLC grade methanol (10 mg/mL) in an ultrasonic bath (Thornton, Brazil) and were passed through a 0.45 μm filter (Millipore, USA). Aliquots of 20 μL were injected onto a C18 column (4.6 x 150 mm, 5 μm particle size, Phenomenex Luna, Torrance, USA). The mobile phase was acetonitrile:phosphoric acid 0.25% (9:1), pH 3.0, which was pumped at a constant flow rate of 1 mL/min (isocratic elution). The eluates were monitored at 210 nm. Further details of extraction, standardization and phytochemical screening have been previously published [10].

### Animals

**Fig 1:**
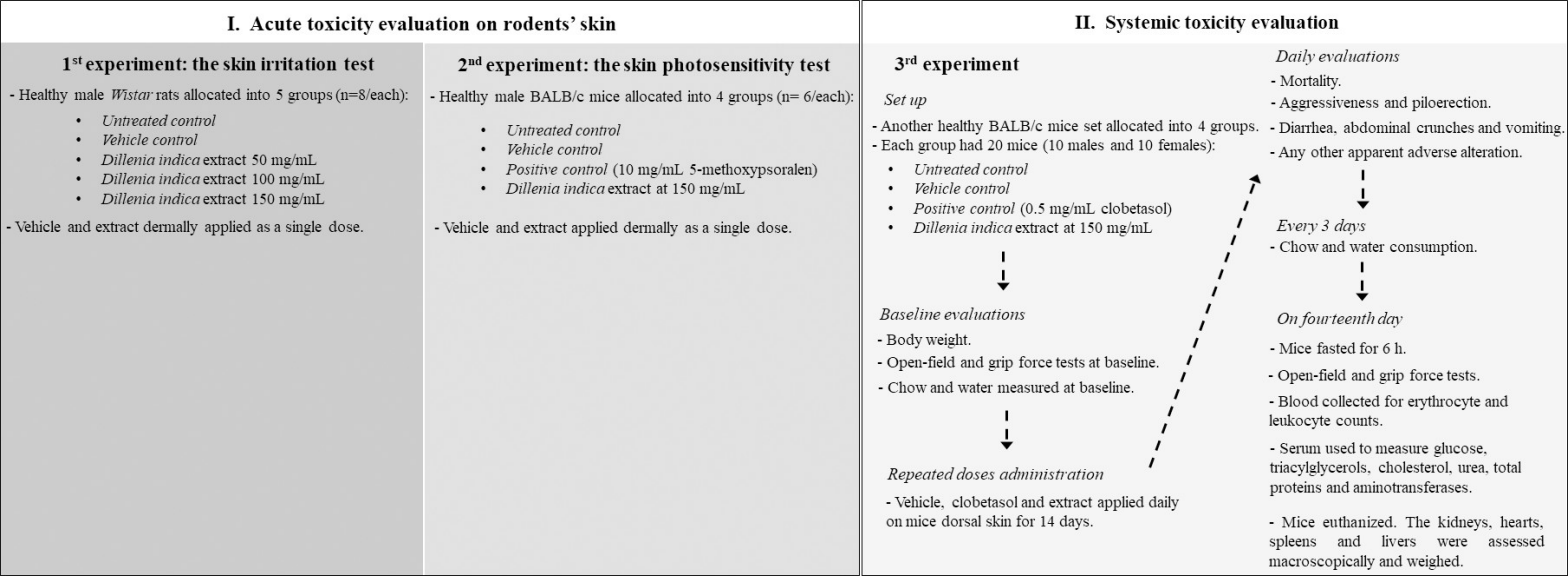
Study design for evaluating the potential adverse effects caused by *Dillenia indica* L. fruit extract standardized to betulinic acid and dermally applied to healthy rodents.

The experiments were performed after the study design (Fig 1) approval by the Ethics Committee of Unisul (14.020.4.03. IV and 15.041.4.01.IV). The animals were used following the internationally accepted principles for laboratory animal use and care (NIH publication #8023, revised in 1978). Two rodent species were included in the study. First, healthy young adult male *Wistar* rats (age close to 8 weeks), weighing 250 ± 25 g, were obtained from Universidade do Vale do Itajaí, Brazil. Second, *Mus muscullus* BALB/c mice were healthy males and females that were donated by the Universidade Federal de Santa Catarina, Brazil. The mice were young adults, aged close to 60 days, and weighed 20 ± 2 g. The animals were housed in separate rooms under controlled conditions (12-hour dark and light cycle, temperature 22 ± 2°C, approximately 60% relative humidity).

### Study design

This study design is summarized in Fig 1. This design corresponded to those used for toxicological evaluations for the development of a drug for dermal administration. The study was carried out in two main phases (I and II) across three different experiments. In the initial phase, the potential adverse acute effects were evaluated locally on the skin application site of a single dose of extract containing betulinic acid. In this phase, a skin irritation test (Fig 1, 1^st^ experiment) and a photosensitivity test (Fig 1, 2^nd^ experiment) were performed. In a subsequent phase (Fig 1, 3^rd^ experiment), the potential systemic toxic alterations caused by repeated doses of extract were evaluated. The application site was always a well-defined depilated skin area on the backs of rodents. The experiments were articulated respecting the guides for the conduction of nonclinical studies of toxicology and the pharmacological safety of the Brazilian Sanitary Surveillance Agency [22,23]; these guidelines are in line with those of drug surveillance agencies (Food and Drug Administration and European Medicines Agency) and with those of institutions with interests in the field (International Conference on Harmonisation of Technical Requirements for Registration of Pharmaceuticals for Human Use, National Cancer Institute, and World Health Organization).

To begin (Fig 1, 1^st^ experiment), the skin irritation test was performed using male *Wistar* rats allocated into five groups, including one untreated control group and one vehicle control group. Initially, three concentrations of extract were tested (50, 100 and 150 mg/mL). Betulinic acid corresponded to 0.5, 1.0 and 1.5%, respectively. The lowest concentration (50 mg/mL) was the same as that previously used in the study in which we showed the antipsoriatic effect of the extract [10]. For the current toxicological study, another group received the extract at the maximum feasible concentration (150 mg/mL). At higher concentrations, it was too difficult to dissolve the extract in the vehicle (ethanol:water 1:5). The last group received the extract at 100 mg/mL, an intermediate concentration. According to the results of this experiment, only the 150 mg/mL concentration was used in the subsequent experiments to reduce the number of animals required.

### First experiment: The skin irritation test

The skin irritation test was performed as previously described [24–26]. Twenty-four hours before the test, two sites on the backs of rats were depilated (2 cm^2^/each). Then, ten minutes before the test, one of these sites was slightly scratched using a sterile blade to induce minimum skin surface irritation. No severe damage or bleeding was caused. The extract and vehicle were applied dermally on both sites and were allowed to stay on the scratched and intact skin for 4 h under occluded patches. The substances were then removed by washing with water. Upon removal, erythema and edema scoring was performed at 1 h, 24 h, 72 h and 7 days later according to the Federal Hazardous Substances Act of the USA CFR 1500.41 [27]. Erythema and edema were graduated through a zero-four scale as follows: zero–no erythema/edema; one–very slight (barely perceptible); two–well defined; three–moderate to severe; and four–severe (beet redness erythema to eschar formations/edema raised more than 1 mm and extending beyond the exposure area). An Irritation Index (I.I.) was calculated according to the following equation [25]: I.I. = (Ʃ erythema grades at 1 h., 24 h., 72 h and day 7 + Ʃ edema grades at 1 h., 24 h., 72 h and day 7)/(number of rats x number of application skin sites x reading times).

### Second experiment: The photosensitivity test

**Fig 2:**
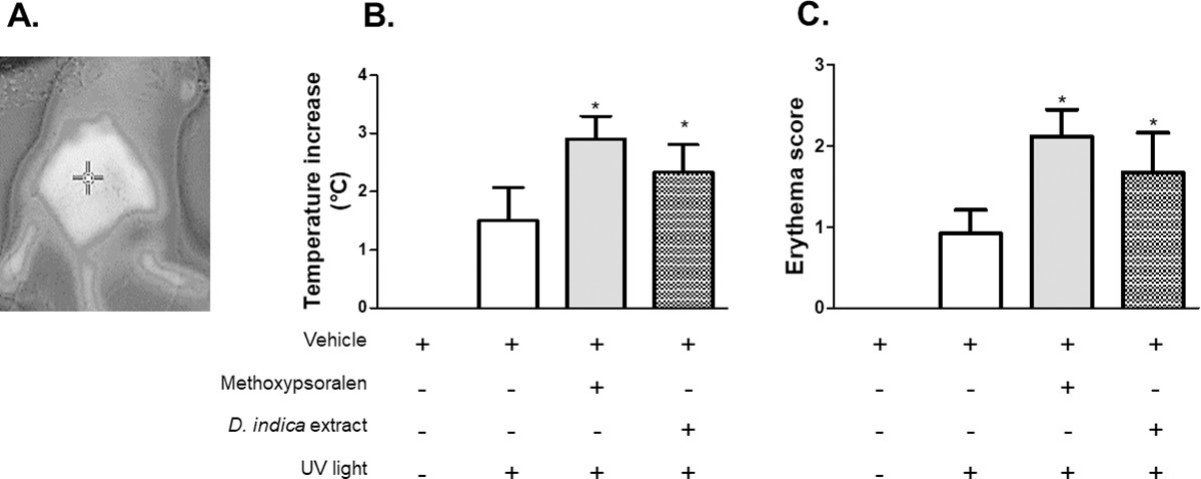
Evaluation data of the photosensitivity-inducing potential of standardized *Dillenia indica* fruit extract (1.5% betulinic acid) (single dose) dermally administered at 150 mg/mL (100 μL) on a shaved area of the back of healthy male BALB/c mice (n = 6/group). Only vehicle (ethanol:water 1:5, 100 μL) was administered to the mice from the untreated and the vehicle control groups, whereas 10 mg/mL 5-methoxypsoralen was applied to the mice in the positive control group. Thirty minutes later, the skin application sites were irradiated with ultraviolet light at a sufficient intensity to cause minimal erythema (1.5 J/cm^2^ at 20 cm for 20 min). Any erythema increase was evaluated 24 h after irradiation. Photosensitization was evaluated with infrared thermography (data in B) performed in the same skin area (as that shown in A) and was evaluated by erythema scoring (data in C). The data were analyzed by the analysis of variance (ANOVA) and the Bonferroni test. *denotes a significant difference in comparison to the vehicle control group (*p <* 0.05).

The photosensitivity test (Fig 1, 2^nd^ experiment) was performed as previously described [28–32]. Twenty-four hours before the test, one central area on the backs of healthy BALB/c male mice (4 cm^2^/each) was shaved. The mice were allocated into four groups (n = 6), including one untreated control group and one vehicle control group, which dermally received a single dose of the vehicle (100 μL ethanol:water 1:5). The positive control group received a dose of a photosensitizer (10 mg/mL 5-methoxypsoralen) [33]. Another group received a single dose of extract (150 mg/mL). Thirty minutes later, the application sites were irradiated for 20 min with UV light (UV lamps, Phillips, Brazil) 20 cm away from the animals’ skin surface, except for the sites of the untreated control group. Irradiation was set at 1.5 J/cm^2^ based on the results of a test performed previously for determining the light intensity capable of inducing a minimal erythema (MED). A photosensitivity skin reaction was evaluated 24 h after irradiation. Erythema was graded with two techniques. The first one was the same zero-four scale described previously [27], and the second one was infrared thermography [34]. Infrared thermography was used under strict room temperature control conditions (22°C). One point to measure the skin temperature was defined in the most central region of the mouse back, as shown in Fig 2A. The digital thermography camera (FLIR, China) was equipped with a 320 x 240 pixel detector matrix of thermal definition < 0.1°C, infrared range: 3.6–5.0 μm. Thermograms were recorded at the baseline and 24 h after irradiation using a wide-angle lens. All analyzed skin regions were photographed in technical triplicates. Since the focus was only the skin temperature changes, any absolute constant error did not significantly affect the results.

### Third experiment: Systemic toxicity evaluation through repeated doses

Healthy BALB/c mice were allocated into four groups (each one had 10 male and 10 female mice kept in separate cages) [22]. The control groups included one untreated group, one vehicle control group that received only the vehicle (100 μL ethanol:water 1:5), and one positive control group that received 0.5 mg/mL clobetasol propionate [35]. Clobetasol is a corticosteroid anti-inflammatory drug that is used to treat psoriasis [36]. The fourth group received the extract at 150 mg/mL (Fig 1, 3^rd^ experiment). Dermal applications were performed every 24 h for 14 consecutive days. During the 14-day treatment period, mortality was monitored daily [37]. After the applications, aggressiveness and piloerection, gastrointestinal changes (diarrhea, abdominal crunches and vomiting) and any other apparent adverse alterations were monitored for two hours [38–40]. In the beginning (baseline) and at the end (1 h after the last dose), the open-field test [41] and the grip force test [42] were performed. The open-field arena (Insight, Brazil) consisted of a polyvinyl chloride box measuring 40 x 60 x 50 cm (height x length x width). The arena floor was divided into 12 equal squares. To evaluate the mice ambulation pattern, the number of squares crossed by the animal (all paws) was counted in a 6-min session. The grip strength test takes advantage of the animal’s tendency to grasp a grid with the front paws while suspended by its tail. The wire frame (10 x 12 cm) was attached to a force transducer (Columbus Instruments, USA). The researcher moved the animal in a rostrocaudal direction until the grip was broken. The force produced during the pull on the bar was repeatedly measured (three times) at intervals with 5 min of rest. The grip force was recorded as the highest value obtained from the three consecutive trials. Changes in the mouse’s nutrition status were monitored by body weight and chow and water consumption. The mice were weighed with an electronic balance (Sartorius, Germany) at the baseline and 1 h after the application of the last dose of extract or the control. To monitor the food and water consumption, the chow was weighed in the same electronic balance, whereas the water volume in the feeders was determined using graduated beakers (Dist, Brazil) [39,40].

#### Collection and preparation of the blood samples and organs

Two hours after the administration of the fourteenth dose of extract and the control, blood samples were collected by retro-orbital puncture. Before the puncture, mice were fasted for 6 h [43,44]. A drop of blood was smeared onto glass slides to prepare thin blood smears, which were stained with the Romanowsky method using the Panotico staining kit (Laborclin, Brazil) [45–47]. The remaining amount of blood was divided between two aliquots in conical microtubes (Axygen, USA). EDTA (1 mg/mL) was homogenized in the first aliquot [48]. The second aliquot was incubated for 30 min in a thermostatic water bath (Fanem, Brazil) at 37°C. After coagulation, this aliquot was centrifuged (10 min/ 5,000 *g*) to obtain the serum [46,47]. Right after, the mice were euthanized, and the kidneys, hearts, spleens and livers were morphologically assessed and then dissected, washed with PBS, dried in absorbent paper and immediately weighed in an analytical balance (Shimadzu AUY 220, Japan) [40].

#### Hematology

Absolute counts of erythrocytes and leukocytes were performed in Neubauer chambers (Kasvi, Brazil) under light microscopy (Olympus microscope, Japan). For erythrocytes, 20 μL of whole blood was diluted in 4 mL of Dacie’s diluent fluid (1 mL of 40% formalin and 99 mL of 3% trisodium citrate). After homogenization, 20 μL of the mixture was used to load the chamber. The cells were counted in five quadrants among the 25 quadrants of the central chamber. The number of red blood cells was calculated by the following equation [48]: erythrocytes (number/mm^3^) = mean erythrocyte count x 10,000. For absolute leukocyte count, a 50 μL aliquot of whole blood was diluted in 950 μL of Turk's solution (1 mL of gentian violet solution and 100 mL of 2% acetic acid solution). After homogenization, approximately 20 μL of the mixture was again used to load the chamber, and the cells were counted in the four peripheral quadrants of the chamber. The number of leukocytes was calculated by the following equation [48,49]: leukocytes (number/mm^3^) = mean leukocyte count x 50. To carry out the differential leukocyte count, one hundred cells were evaluated per slide, and each slide corresponded to the blood sample of one animal. Leukocytes were classified under light microscopy according to the morphological characteristics of lymphocytes, monocytes, neutrophils, eosinophils and basophils [48,49]. The erythrocyte sedimentation rate (ESR) was measured in graduated glass tubes filled with whole blood and left in upright position for 1 h. ESR was read on the descending scale of each tube [50]. The microhematocrit technique was used to determine the hematocrit [51]. Capillary tubes (1.2 x 75 mm) containing anticoagulant were filled with whole blood up to two-thirds of their volume, and each tube was sealed at one end. Then, the tubes were centrifuged (10,000 x *g* for 5 min). The data were obtained on a 0 to 100 mm scale, observing the limit of separation of the erythrocyte mass from the plasma. The results were expressed as a percentage of erythrocytes in relation to the total blood volume (mean ± standard deviation). Finally, the mean corpuscular volume (MCV) was calculated with the following equation [52,53]: MCV (fL) = hematocrit (%) x 10/erythrocyte number per mm^3^.

#### Serological analyses

These evaluations were performed with spectrophotometry (Perlong Microplate Reader, China), using commercially available kits (Labtest, Brazil), and the protocols suggested by the manufacturer were followed. The serum concentrations of glucose, triglycerides, cholesterol, urea, total protein content and the activities of aspartate aminotransferase (AST) and alanine aminotransferase (ALT) were measured. The measurements were performed in technical triplicates. The data are shown as the means ± standard deviations.

### Statistical analyses

Power analyses indicated that at least six animals per group were needed to detect differences in skin irritation and photosensitivity with a power of 0.80 and an α probability of 0.05. For the repeated doses toxicity study, the number of animals per group was determined according to the referred guides for the conduction of nonclinical studies of toxicology and pharmacological safety [22]. The data are shown as the means ± standard deviations. The normal data distribution was evaluated by the Shapiro-Wilk test. Then, the data were subjected to analysis of variance (ANOVA) and the Bonferroni test. Comparisons were performed using GraphPad Prism software (San Diego, USA) according to the animal’s sex. Values of p ≤ 0.05 were considered statistically significant.

## Results

### Acute effects on skin

The data in Table 1 correspond to the skin irritation index, which was calculated and interpreted along with the reference values from the literature [24]. Accordingly, the extract was considered to be nonirritating to the skin of healthy rats. Table 1 shows that even the irritation index corresponding to the extract applied at the highest concentration of 150 mg/mL (1.5% betulinic acid) was rated as causing negligible irritation.

**Table 1:** Values of the irritation index determined in healthy male *Wistar* rats (n = 8/each).

Groups	Irritation Index
- Untreated control group	0.00
- Vehicle control group	0.10
- Extract treated groups:	
* *Dillenia indica* extract 50 mg/mL	0.09
* *Dillenia indica* extract 100 mg/mL	0.18
* *Dillenia indica* extract 150 mg/mL	0.28

The vehicle control group was treated only with the vehicle (ethanol:water 1:5), whereas the test groups received the standardized extract from *Dillenia indica* fruit at the concentrations indicated in the table (0.5–1.5% betulinic acid, respectively). The treatments were dermally applied with a single dose on the backs of healthy male animals, which were previously lightly scratched with a scalpel blade. The irritation index was derived from scoring erythema and edema at 1 h, 24 h, 72 h and 7 days after the treatments according to the Federal Hazardous Substances Act of the USA CFR 1500.41.

* 0.5; 1.0 and 1.5% betulinic acid in the extract, respectively. Irritation Index descriptive rating [24]: Negligible irritation (0–0.4); Slight irritation (> 0.5–1.9); Moderate irritation (2.0–4.9); Severe irritation (> 5.0).

The data in Fig 2 are the results of the experiment performed to verify whether the extract was capable of inducing skin photosensitization in mice. The data in Fig 2B and Fig 2C show that the skin of mice from the vehicle control group, which were exposed to ultraviolet light at MED, had a mild increase in temperature and erythema. The photosensitization caused by 5-methoxypsoralen (10 mg/mL) was confirmed by the increased temperature (an average of 2°C) and increased erythema (> 2-fold) compared to the temperature and erythema in the vehicle control group. The data suggest that the extract acted as a photosensitizer because the temperature and erythema of the skin treated with extract were significantly higher (up to 2-fold) compared to those of the vehicle control group.

### Systemic effects from repeated doses

No mortality occured during the long-term experiment of extract systemic toxicity in any animal group (male or female mice). Repeated doses of extract applied to the intact skin of healthy mice caused no changes in aggression patterns, piloerection occurrence, gastrointestinal changes (diarrhea, vomiting, and abdominal crunches) or convulsions.

The data in Table 2 correspond to the indicators used to assess impairments in mice’s nutrition status (weight gain and water and chow consumption). No significant change was observed between the data of mice receiving the extract and mice of the control groups. Table 3 shows the average data and standard deviations corresponding to the mean weights of the mouse kidneys, hearts, spleens and livers. Macroscopically, the organ morphology did not change after the treatments. The statistical analyses carried out according to sex did not show any differences when the data of mice treated with extract were compared to the data of the control groups mice.

**Table 2:** Data of healthy BALB/c mice (n = 20, male:female 1:1) concerning the nutrition *status*.

	Male mice treatments					Female mice treatments				
Parameter	Untreated	Vehicle	Clobetasol	*D*. *indica*	*p* value	Untreated	Vehicle	Clobetasol	*D*. *indica*	*p* value
Weight gain (g)	1.2 ± 1.0	0.8 ± 0.6	1.3±1.2	1.3 ± 1.2	0.66	0.2 ± 0.3	0.1 ± 0.1	0.2 ± 0.4	0.1 ± 0.1	0.69
Water consumption (mL)	59.9 ± 9.0	76.6 ± 20.2	60.0 ± 10.0	76.6 ± 41.9	0.21	57.0 ± 18.5	60.0 ± 26.4	56.6 ± 16.0	56.6 ± 20.8	0.98
Chow consumption (g)	82.5 ± 10.0	88.3 ± 13.6	80.6 ± 11.0	83.0 ± 11.3	0.49	70.5 ± 40.0	67.6 ± 28.0	75.6 ± 46.2	80.6 ± 49.6	0.90

Animals were treated dermally every 24 h for 14 consecutive days on an intact skin area (4 cm^2^) on their backs. One group received standardized *Dillenia indica* fruit extract (1.5% betulinic acid) at 150 mg/mL (100 μL), whereas 0.5 mg/mL clobetasol was administered to mice from the positive control group. Only the vehicle (100 μL ethanol:water 1:5) was applied to mice from the vehicle control group. The data are the means ± standard deviations and were analyzed by the analysis of variance test (ANOVA). No significant differences were verified among the data.

**Table 3:** Weight of the organs of healthy BALB/c mice (n = 20, male:female 1:1), which were treated dermally every 24 h for 14 consecutive days on an intact skin area (4 cm^2^) on their backs.

	Male mice treatments					Female mice treatments				
Organs	Untreated	Vehicle	Clobetasol	*D*. *indica*	*p* value	Untreated	Vehicle	Clobetasol	*D*. *indica*	*p* value
Kidneys (g)	570 ± 40	540 ± 60	550 ± 30	580 ± 30	0.15	350 ± 30	340 ± 40	340 ± 30	360 ± 35	0.50
Heart (g)	145 ± 10	140 ± 10	150 ± 10	140 ± 10	0.09	115 ± 15	110 ± 15	120 ± 10	110 ± 10	0.26
Spleen (g)	85 ± 25	80 ± 30	70 ± 10	90 ± 20	0.25	55 ± 15	60 ± 20	50 ± 15	60 ± 10	0.42
Liver (g)	1490 ± 110	1510 ± 100	1495 ± 100	1500 ± 120	0.98	1130 ± 210	1170 ± 200	1140 ± 220	1120 ± 100	0.94

Standardized *Dillenia indica* fruit extract (1.5% betulinic acid) was administered at 150 mg/mL (100 μL), whereas 0.5 mg/mL (100 μL) clobetasol was administered to the mice from the positive control group. Only the vehicle (100 μL ethanol:water 1:5) was administered to mice from the vehicle control group. The data are the means ± standard deviations and were analyzed by analysis of variance (ANOVA). No significant differences were verified among the data.

**Fig 3:**
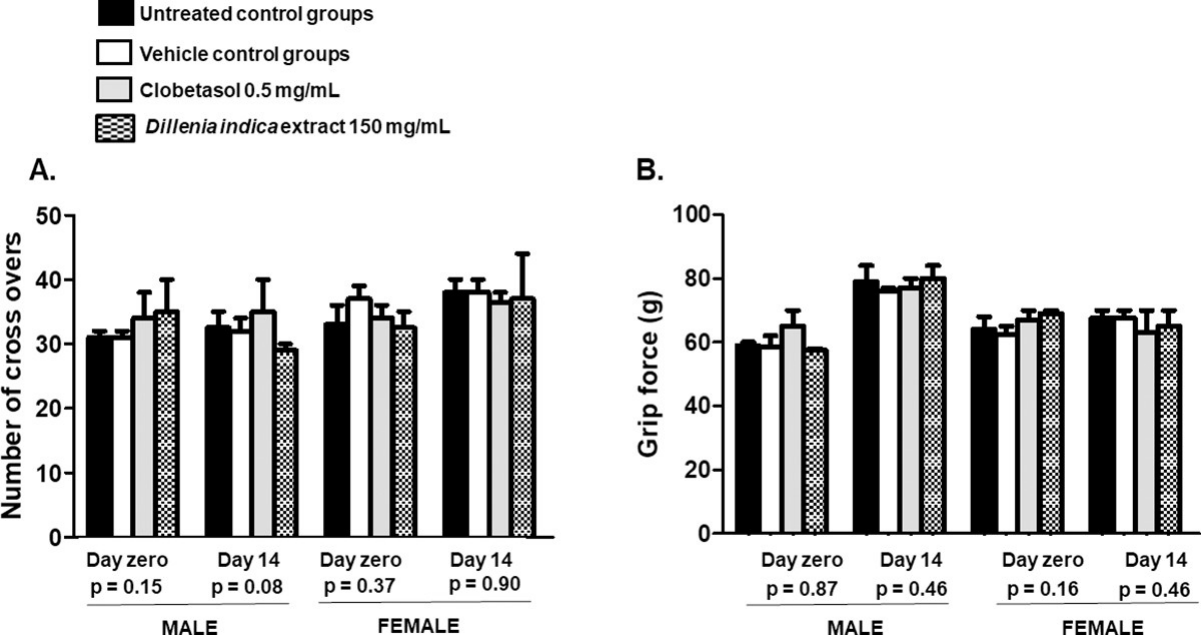
**Number or crossovers representing the average ambulation behavior (A) and grip force (B) of mice from both sexes.** The mice were healthy BALB/c mice (n = 20, male:female 1:1), which were dermally treated every 24 h for 14 consecutive days on an intact skin area (4 cm^2^) on their backs. One group received standardized *Dillenia indica* fruit extract (1.5% betulinic acid) at 150 mg/mL (100 μL), whereas 0.5 mg/mL clobetasol was administered to the mice from the positive control group. Only the vehicle (100 μL ethanol:water 1:5) was administered to the mice from the vehicle control group. The data were analyzed by analysis of variance (ANOVA). No significant differences were verified among the data.

Evidence of the integrity of motor and neurological activities is shown by the data from the open-field and grip force tests in Fig 3A and Fig 3B, respectively. No significant changes were observed in terms of ambulation or grip force when comparing the data from the male or female mice receiving the extract to the data from the male or female mice from the control groups.

The data of hematological parameters are shown in Table 4. The mean hematological profiles of healthy male and female mice may be seen with the data from the untreated control groups. There were no significant differences between the mice receiving the extract and the mice receiving only the vehicle. On the other hand, the comparisons between the data of the positive control (clobetasol) groups and the data of the vehicle control groups revealed some significant differences in certain leukocyte counts. Apparently, repeated clobetasol application on intact skin (at the dosage used) caused a reduction in the absolute leukocyte number along with a reduction of the lymphocytes and monocytes in the blood samples of male and female mice. The data resulting from serum assessments are shown in Table 5, and again, no significant differences were observed in the parameters examined in the mice receiving the extract and the mice of the control groups.

**Table 4:** Hematological data of healthy BALB/c mice (n = 20, male:female 1:1), which were dermally treated every 24 h for 14 consecutive days on an intact skin area (4 cm^2^) on their backs.

Hematological parameters	Male mice treatments					Female mice treatments				
	Untreated	Vehicle	Clobetasol	*D*. *indica*	*p* value	Untreated	Vehicle	Clobetasol	*D*. *indica*	*p* value
Red blood cells (millions/mm^3^)	7.1 ± 1.0	7.0 ± 0.8	6.8 ± 0.6	7.2 ± 1.1	0.78	5.8 ± 0.5	5.6 ± 0.4	5.7 ± 0.5	6.0 ± 0.6	0.34
Leukocytes (thousands/mm^3^)	3.8 ± 0.8	4.0 ± 1.1	1.4 ± 0.9*	3.5 ± 0.6	< 0.001	3.0 ± 0.8	3.6 ± 0.8	1.7± 0.5*	3.5 ± 0.9	< 0.001
Neutrophils (%)	75.0 ± 8.0	70.0 ± 9.0	80.0 ± 10.0	76.0 ± 7.0	0.09	71.0 ± 4.5	74.0 ± 5.0	73.0 ± 3.0	70.0 ± 5.0	0.19
Lymphocytes (%)	20 ± 4.5	25.0 ± 5.0	12.0 ± 3.0*	22.0 ± 3.0	< 0.001	25.0 ± 3.5	23.0 ± 4.0	11.0 ± 4.0*	28.0 ± 2.0	< 0.001
Monocytes (%)	8.0 ± 1.9	7.0 ± 2.0	3.0 ± 2.0*	9.0 ± 2.0	< 0.001	4.5 ± 1.0	5.0 ± 2.0	2.0 ± 2.0*	4.0 ± 1.0	0.0009
Eosinophils (%)	1.0 ± 1.0	1.0 ±1.0	1.0 ± 1.0	1.0 ± 1.0	1.0	0	0	0	0	0
Basophils (%)	0	0	0	0	0	0	0	0	0	0
ESR (mm/h)	5.0 ± 2.0	5.0 ± 2.0	5.0 ± 2.0	5.0 ± 2.0	1.00	5.0 ± 2.0	5.0 ± 2.0	5.0 ± 2.0	5.0 ± 2.0	1.00
Hematocrit (%)	60 ± 5.0	61.8 ± 5.6	58.2 ± 2.6	61.2 ± 1.7	0.22	56.0 ± 6.5	58.4 ± 5.3	54.2 ± 6.3	59.2 ± 7.6	0.30
MCV (fL)	80.0 ± 9.0	83.4 ± 6.6	79.7 ± 10.5	82.2 ± 6.9	0.72	79.5 ± 5.5	80.2 ± 11.0	76.5 ± 6.5	76.5 ± 2.8	0.52

Standardized *Dillenia indica* fruit extract (1.5% betulinic acid) was administered at 150 mg/mL (100 μL), whereas 0.5 mg/mL (100 μL) clobetasol was administered to the mice from the positive control group. Only the vehicle (100 μL ethanol:water 1:5) was applied to the mice from the vehicle control group. Erythrocyte sedimentation rate (ESR) and mean corpuscular volume (MCV). The data are the means ± standard deviations and were analyzed by analysis of variance (ANOVA) and the Bonferroni test.

* denotes a significant difference compared to the vehicle control group (*p* < 0.05).

**Table 5:** Serological data of healthy BALB/c mice (n = 20, male:female 1:1) that were dermally treated every 24 h for 14 consecutive days on an intact skin area (4 cm^2^) on their backs.

Serum parameters	Male mice treatments					Female mice treatments				
	Untreated	Vehicle	Clobetasol	*D*. *indica*	*p* value	Untreated	Vehicle	Clobetasol	*D*. *indica*	*p* value
Glycemia (mg/dL)	64.1 ± 13.0	67.6 ± 8.0	57.6 ± 12.0	63.2 ± 13.0	0.30	70.5 ±15.0	69.5 ± 9.4	74.6 ± 15.4	75.2 ± 16.6	0.75
Cholesterol (mg/dL)	96.2 ± 9.0	95.3 ± 14.0	95.3 ± 14.8	83.2 ± 27.0	0.30	90.0 ±16.0	95.8 ± 18.5	95.8 ± 18.6	86.4 ± 16.6	0.56
Triglycerides (mg/dL)	105.0 ± 35.0	108.3 ±35.0	90.3 ± 35.8	104.0 ± 35.0	0.68	155.5 ±70.0	164.2 ± 80.4	144.4 ± 42.1	192.0 ± 63.0	0.42
Urea (mg/dL)	36.0 ± 11.0	37.6 ± 7.8	37.7 ± 13.4	33.2 ± 13.2	0.80	35.0 ± 11.0	27.6 ± 7.8	37.7 ± 13.4	33.2 ± 13.2	0.27
Total protein (g/dL)	6.9 ± 0.3	7.0 ± 0.2	6.8 ± 0.5	7.0 ± 0.5	0.63	7.0 ± 0.8	6.9 ± 0.4	7.1 ± 0.5	6.5 ± 1.0	0.27
AST (U)	25.5 ± 7.0	28.6 ± 11.0	30.0 ±8.0	22.8 ± 3.0	0.18	25.0 ± 11.0	27.1 ± 18.1	23.0 ± 10.0	23.0 ± 5.4	0.85
ALT (U)	33.0 ± 10.0	36.2 ± 8.0	35.0 ± 10.0	31.0 ± 18.0	0.78	30.0 ± 8.0	32.1 ± 4.5	28.0 ± 10.0	31.2 ± 7.4	0.67

Standardized *Dillenia indica* fruit extract (1.5% betulinic acid) was administered at 150 mg/mL (100 μL), whereas 0.5 mg/mL (100 μL) clobetasol was administered to the mice from the positive control group. Only the vehicle (100 μL ethanol:water 1:5) was applied to mice from the vehicle control group. AST: Aspartate aminotransferase. ALT: Alanine aminotransferase. The data are the means ± standard deviations and were analyzed by analysis of variance (ANOVA). No significant differences were verified among the data.

## Discussion

The research question approached by this work was based on several previous studies that showed the beneficial bioactivities of *D*. *indica* extracts and derivatives. Betulinic acid, a major constituent of *D*. *indica* extract, has been extensively studied in recent years. This molecule was mostly studied for its benefits. Most previously conducted toxicological studies on betulinic acid used cytotoxicity assays in cell culture and focused on antitumor activities [54,55].

In fact, betulinic acid may be abundant in other plants, such as *Zizyphus joazeiro* [56], *Syzigium clariflorum* [57], and others. There are many studies that have demonstrated the anti-inflammatory properties of betulinic acid, and there is an assortment of studies showing its potential toxicity to bacteria, protozoa, helminths, fungi and tumor cells. Betulinic acid’s antiviral and antitumor potentials are noteworthy [54,55,57–59]. Previously, phase I/II clinical trials have evaluated 20% betulinic acid ointments for the safety and efficacy of preventing dysplastic nevi from progressing into melanomas (http://clinicaltrials.gov/ct2/show/NCT00346502). Another clinical trial is evaluating the safety, tolerability, and preliminary betulinic acid efficacy in patients with cutaneous metastatic melanoma (https://clinicaltrials.gov/ct2/show/NCT00701987). However, no results have been posted on ClinicalTrials.gov thus far. Therefore, the data of the current work may contribute to optimizing the designs of future studies and may help to define the dose, reduce the risks and refine clinical trials.

The data from this study corresponded to the administration of *D*. *indica* fruit extract at concentrations of up to 150 mg/mL (1.5% betulinic acid) on the skin of healthy rodents. In terms of acute and local effects, the data of the skin irritation test demonstrate that *D*. *indica* fruit extract does not have an irritative potential. In fact, the nonirritative effect of this product was evident during the whole treatment period with repeated doses. On the other hand, the data suggest that the extract acted as a photosensitizer.

The data in Fig 2B and Fig 2C show that the administration of the extract induced skin photosensitivity in mice with an intensity that was close to that of 5-methoxypsoralen (10 mg/mL). This finding does not necessarily represent a limitation to prevent this extract from becoming a component in a future formulation for treatment by dermal application. Obviously, this information would have to be given along with proper use criteria and instructions. Several other commercially available drugs have this feature as well; for example, dapsone, isotretinoin, acitretin, and other drugs, including some antipsoriatic drugs, are known to induce photosensitivity when applied dermally [60–62]. Patients using these drugs must be informed about this side effect and must use these drugs with caution. From another point of view, the photosensitizer activity may eventually be useful for phototherapy. For this type of therapy, a combination of light, oxygen and a photosensitizer is used [63]. This combination is widely practiced in dermatology [64,65]. It is particularly useful for patients with multiple, disseminated superficial lesions and for immunosuppressed patients. It has been considered a safe option for the treatment of acne vulgaris, psoriasis, viral warts, local scleroderma, photoaging, leishmaniasis, and other dermatological disorders [65]. Therefore, it is reasonable to hypothesize that the fruit extract from *D*. *indica* may be a promising bivalent compound with healing activities [10], and eventually, this extract may be used in phototherapy.

Since the treatments were performed with the extract and not from isolated substances, it was not possible to determine which particular constituent was responsible for the photosensitizing effect. However, the phototoxic effects of betulinic acid had been previously evaluated, and no photosensitizing effects were reported for betulinic acid extracted from *Stachytarpheta cayennensis* (Rich.) Vahl, Verbenaceae [66]. Moreover, there is the possibility of light reactions with some of the flavonoids in the extract. Flavonoids are ubiquitous in photosynthesizing cells [67]. One previous study has shown that the natural flavonoid silybin improved the response of bladder cancer cells to photodynamic therapy [68].

Afterwards, the evaluations were focused on the systemic consequences from repeated extract administration on the intact skin of healthy mice. This type of evaluation is one of the first steps to assess the safety of a potential new drug [22]. Preclinical toxicity tests are necessary to determine the level of nonobserved adverse side effects for a particular compound, which is required before future studies and clinical trials can begin [69]. In the current study, the toxicity was evaluated with a study that was designed (Fig 1, 3^rd^ experiment) to enable the identification of systemic effects in the following systems: the nervous, digestive, hematopoietic, immunological, cardiac, hepatic and renal systems.

Mortality was the first toxicity endpoint assessed. Through this evaluation, it is possible to calculate the lethal dose 50 (LD50), which is defined as the dose that causes the death of 50% of the individuals in the experimental treatment group. No mortality was observed in this study; therefore, an LD50 calculation was not possible. In fact, the data indicated that repeated extract administration (on the intact skin of healthy mice) at 150 mg/mL did not cause any observable adverse effects.

Along with mortality, the mice were monitored during and after the treatment for the occurrence of aggressiveness, piloerection, diarrhea, vomiting, abdominal spasms and convulsions. These are indicators that represent the disruption of neurological and gastrointestinal integrity. Neurological changes can be caused by chemicals present in some plants. These changes may appear in the form of cardiovascular, respiratory, metabolic and/or gastrointestinal disorders; in addition, these disorders may include several other symptoms, such as salivation, vomiting, seizures, coma and even death [70,71]. Other symptoms include decreased consciousness, sudden changes in behavior, lethargy, tremors, muscular atrophy, and others [72]. None of these alterations were observed in the mice receiving repeated extract doses on their intact skin. Further evidence about the integrity of the neurological and motor systems was provided by the data from the open-field and grip force tests. These tests evaluate ambulation and muscular force, respectively (Fig 3A and Fig 3B).

Some plants may contain toxins that can cause toxic effects and may appear as dyspepsia, decreased food consumption, weight loss and dehydration. It is known that this type of intoxication can culminate in growth impairment and immune system suppression [73,74]. The nutritional status may be evaluated through several approaches, such as physical examinations (which determine physical alterations, such as weight gain or loss) and laboratory results (which may detect nutritional problems at early stages) [75]. The data in Table 2 corresponded to the evaluations of this kind. This monitoring was complemented by data of the serum protein concentrations (Table 5), which were also not affected. None of these data suggest that the mice receiving the extract were nutritional compromised.

In toxicity studies, vital organs must be weighed, and this serves as an important indicator to assess the potential toxicity of a compound. This includes the animal's body mass monitoring [76,77]. The kidneys and liver are among the major xenobiotic biotransformation organs [78–80]. In the case of the kidneys, since these organs are the main chemical excretion route, they are the most likely organ to exhibit any chemically-induced toxic effects [81]. The data in Table 3 suggest that in both male and female mice, no significant differences were found in the weights of the kidneys, hearts, spleens and livers.

In animal toxicological studies, blood is the most commonly used sample [82]. A blood test may yield information about the blood itself, the bone marrow or other organs and tissues that are perfused by the blood [83–85]. In principle, a typical toxicological blood test includes a basic metabolic panel (biochemical analyses) and a complete blood count [82,86,87]. The data for these analyses are shown in Table 4 and Table 5.

In toxicology, absolute erythrocyte counts can provide clues related to the toxic effects occurring on the red blood cell membranes, which can result in hemolysis. Compounds with promising biological activities may be rendered useless if they cause hemolysis [88]. A reduction in the red blood cell count is also observed in cases of bone marrow suppression, when pancytopenia may be expected [89]. The abnormal morphology of red blood cells can be inferred through the MCV. An increased MCV value may indicate anemia, acute bleeding, myelodysplastic syndromes, folic acid or vitamin B12 deficiencies. A decreased MCV value may indicate iron deficiency, hyperuremia, severe chronic intoxication, and other harmful effects [90]. ESR is a common hematological test and is a nonspecific measure of inflammation [91]. A hematocrit is used to measure the red blood cell proportion in a blood sample. It is usually performed when anemia or polycythemia is suspected. The test may indicate whether there is a problem in erythrocyte production or with the erythrocyte half-life [47,84]. The data in Table 4 representing the assessment of blood cells and related indicators suggest that none of these disturbances occurred in the mice receiving the extract.

Regarding white blood cells, it is known that toxic effects can mainly affect granulocyte proliferation and function. Moreover, some types of leukemia can be triggered from exposure to toxins. Due to their high physiological proliferation rate, neutrophils and their precursors are particularly susceptible to mitosis inhibitors. Toxic effects on granulocytes (myelotoxicity) are commonly observed in response to treatment with DNA synthesis inhibitors [92]. The data in Table 4 show that the mean leucogram of mice treated with *D*. *indica* fruit extract was not significantly different compared to that of the mice of the vehicle control group. There was a difference between the data of the positive control group (treated with clobetasol) and the data of the vehicle control group; this suggests a reduction in the overall leukocyte number and in the mononuclear counts caused by repeated clobetasol treatments. Therefore, although the dose of clobetasol was initially considered to be safe, this dose was actually the safety threshold because reduced numbers of circulating lymphocytes and monocytes were observed in the mice, as seen in Table 4. This effect had been previously reported in the literature [93].

Finally, Table 5 shows the data of the serological evaluations, including the glycemia values. In toxicology, fasting hyperglycemia is used as a marker to identify compounds with toxic effects on pancreatic beta cells [94]. Serum lipid concentrations may provide evidence of toxic effects on the lipid metabolism; if the lipid metabolism is affected, then the liver would be the most likely target of toxicity. Hepatotoxicity is a major cause for drug withdrawal from the market and can lead to changes in lipid biosynthesis and hepatic steatosis [95]. The blood urea concentration is an indicator of renal function and may serve as an index of the glomerular filtration rate [96]. As elevated urea concentration may indicate renal overload, acute renal failure, or even an increase in protein catabolism [97]. In animals treated with *D*. *indica* fruit extract, the unchanged serum urea concentrations reinforced the idea that kidney toxicity did not occur.

The unchanged aminotransferase activity was additional evidence of the integrity of animal organs. Transaminases are widely distributed in tissues. AST predominates in the liver, heart, heart muscle, striated muscle, kidneys and pancreas; however, ALT predominates in the liver, kidneys and heart [98]. ALT is rapidly elevated when the liver is damaged; therefore, ALT is an important marker of drug-induced liver toxicity [99]. AST is a cell damage marker used to evaluate exposure to toxins. It is present in the cytoplasm and mitochondria. Therefore, AST elevation indicates severe cellular damage, which may represent drug-induced necrosis in hepatocytes. In toxicology, when AST values are increased, they may indicate hepatic cirrhosis, drug-induced hepatitis, hemolytic anemia, pancreatitis, and other harmful conditions. [99]. As observed in the current study, the administration of *D*. *indica* fruit extract on the intact skin of mice (1.5% betulinic acid) did not cause alterations in aminotransferase activity.

## Conclusions

The extract caused no irritation on the skin of rats; however, it acted as a photosensitizer on the mouse skin. No systemic toxicity was observed from the administration of repeated doses of extract on the intact skin of healthy mice. In general, the data suggest that the use of *D*. *indica* fruit extract is safe; this is important for cases where the extract may eventually become a constituent of a future dermal application formulation. This photosensitizing effect deserves to be explored further as it may be interesting for the development of drugs used in the phototherapy of some skin diseases. Although this work allows for some inferences to be made about the safety of the dermal administration of betulinic acid, a fair assessment will depend on further studies with the purified compound and an evaluation of betulinic acid administration on wounded skin, which normally has a compromised barrier function. These experiments are in progress in our laboratory.
